# Use of Janus kinase inhibitors during early pregnancy in patients with rheumatic disease: a case series

**DOI:** 10.1016/j.ero.2026.100240

**Published:** 2026-10-22

**Authors:** Ainsley Needham, Vienna Cheng, Jeremiah Tan, Mary A. De Vera, Neda Amiri

**Affiliations:** 1 Arthritis Research Canada, Vancouver, British Columbia, Canada; 2 Faculty of Pharmaceutical Sciences, University of British Columbia, Vancouver, British Columbia, Canada; 3 Collaboration for Outcomes Research and Evaluation, Vancouver, British Columbia, Canada; 4 Division of Rheumatology, Department of Medicine, Faculty of Medicine, University of British Columbia, Vancouver, British Columbia, Canada

## Abstract

**Background:**

Janus kinase inhibitors (JAKi) are increasingly used to treat systemic autoimmune rheumatic diseases in patients of reproductive age, but pregnancy safety data remain limited. Current guidelines recommend discontinuation before conception and avoidance during pregnancy; however, inadvertent early exposures occur and create counselling uncertainty.

**Case summary:**

We identified 3 pregnancies with confirmed postconception JAKi exposure among 111 patients followed in a specialised pregnancy and rheumatic diseases clinic between July 2021 and September 2024. Two patients with psoriatic or seronegative inflammatory arthritis were exposed to upadacitinib 15 mg daily for 16 and 13 days after conception, respectively. One patient with rheumatoid arthritis was exposed to tofacitinib 5 mg twice daily for 30 days after conception. All JAKi were discontinued when pregnancy was recognised. One patient remained off immunosuppression until postpartum flare. One required prednisone and transitioned to certolizumab for a second-trimester inflammatory arthritis flare. One transitioned to hydroxychloroquine with stable disease. All pregnancies resulted in live births at 37 + 6 to 39 weeks. No congenital anomalies were identified. One infant, delivered by emergency caesarean section complicated by difficult fetal extraction, developed respiratory distress at birth and was admitted to the neonatal intensive care unit for 3 days for monitoring. The available documentation did not identify suspected or confirmed infection.

**Conclusions:**

These 3 cases add to limited real-world evidence regarding inadvertent first-trimester JAKi exposure in rheumatic disease. The findings are reassuring but cannot establish safety, and prospective data with longer infant follow-up remain needed.

**WHAT IS ALREADY KNOWN ON THIS TOPIC**

• Janus kinase (JAK) inhibitors are not recommended during pregnancy because human safety data are sparse and animal studies have raised concerns at supratherapeutic exposures.
• Inadvertent early pregnancy exposure occurs as JAK inhibitors are used more often in patients of reproductive age.

**WHAT THIS STUDY ADDS**

• Three pregnancies with brief postconception exposure to upadacitinib or tofacitinib resulted in live births without identified congenital anomalies.
• The cases provide practical clinical context for counselling after inadvertent first-trimester exposure, while emphasising the limits of small descriptive datasets.

**HOW THIS STUDY MIGHT AFFECT RESEARCH, PRACTICE OR POLICY**

• These observations support continued reporting to pregnancy registries and careful, nondirective counselling rather than assumptions of either harm or safety after brief early exposure.

Alt-text: Unlabelled box dummy alt text

## INTRODUCTION

Active systemic autoimmune rheumatic disease during pregnancy is associated with adverse maternal and neonatal outcomes; therefore, disease control before conception and throughout pregnancy remains central to care [1,2]. Janus kinase inhibitors (JAKi), including tofacitinib and upadacitinib, have expanded treatment options for inflammatory arthritis and other immune-mediated diseases [3]. Their use in patients of reproductive age is increasing, but pregnancy data remain limited because pregnant patients are excluded from clinical trials.

JAKi are small molecules that may cross the placenta, including during early embryogenesis [3,4]. Animal studies have reported fetal toxicity and teratogenicity at exposures above those used clinically [5,6]. Current reproductive health guidance recommends discontinuing JAKi before conception and avoiding their use during pregnancy [5,6]. Nevertheless, unplanned pregnancies and delayed pregnancy recognition can lead to inadvertent exposure. Clinicians and patients then require balanced counselling based on limited human evidence.

We report 3 pregnancies with inadvertent first-trimester exposure to upadacitinib or tofacitinib, identified through a specialised pregnancy and rheumatic disease clinic.

## CASE SERIES

Patients were identified from a pregnancy and rheumatic disease registry at the University of British Columbia. The registry includes patients aged ≥18 years with systemic autoimmune rheumatic disease who are pregnant or planning pregnancy. We reviewed patients enrolled between July 2021 and September 2024 and included those with confirmed overlap between pregnancy and JAKi use. Estimated conception dates were based on first-trimester ultrasound and standard obstetric dating. Outcomes were abstracted from clinic notes, electronic medical records, and registry data. The registry did not include systematic longitudinal paediatric follow-up beyond the early postnatal period. Consequently, data regarding vaccine responses, subsequent infections, and developmental milestones were not available.

### Case 1

A 40-year-old G4P3 patient with psoriatic arthritis and polycystic kidney disease conceived while taking upadacitinib 15 mg daily, which had controlled her arthritis for ∼18 months. Her previous pregnancies resulted in 3 term deliveries: 1 caesarean section for dystocia followed by 2 successful vaginal births after caesarean. The index pregnancy was unplanned. Upadacitinib was continued for 16 days after conception and stopped when the pregnancy was recognised. The patient elected to remain off immunosuppressive therapy during pregnancy and had no clinically documented inflammatory arthritis flare. She delivered a healthy infant by elective caesarean section at 37 + 6 weeks. Birth weight was 3160 g, and Apgar scores were 9 and 9 at 1 and 5 minutes, respectively. No congenital anomalies were identified. She resumed upadacitinib 3 months postpartum after breastfeeding had ended, and arthritis symptoms recurred.

### Case 2

A 39-year-old G4P1 patient with seronegative inflammatory arthritis, ulcerative colitis, and central nervous system demyelinating lesions conceived while taking upadacitinib 15 mg daily. Her obstetric history included 1 previous term caesarean delivery; further details regarding the outcomes of her other prior pregnancies were not available in the records reviewed. Upadacitinib had been used for ∼5 months and was continued for 13 days after conception before being discontinued when pregnancy was recognised. She initially remained off alternative immunosuppression but developed an inflammatory arthritis flare in the second trimester. Prednisone 15 mg daily was started. Ixekizumab was attempted without clinical improvement, and certolizumab 200 mg every 2 weeks was subsequently initiated with a good response. Prednisone was tapered to 2.5 mg daily by delivery and discontinued postpartum. She delivered a healthy infant by planned repeat caesarean section at 39 weeks. Birth weight was 3033 g, and Apgar scores were 9 and 9 at 1 and 5 minutes, respectively. No congenital anomalies were identified.

### Case 3

A 40-year-old G2P1 patient with rheumatoid arthritis conceived while taking tofacitinib 5 mg twice daily. Her previous pregnancy was uncomplicated and resulted in a term vacuum-assisted vaginal delivery. She had not been receiving disease-modifying antirheumatic therapy during that pregnancy and did not experience a significant postpartum rheumatoid arthritis flare. She had previously used methotrexate with tofacitinib but had stopped methotrexate before conception because of disease control and personal preference. Tofacitinib exposure continued for 30 days after conception and was stopped when pregnancy was recognised. Hydroxychloroquine 200 mg twice daily was started, and her arthritis remained clinically controlled. At 39 weeks, she underwent an emergency caesarean section for arrest of descent. Delivery was complicated by difficult fetal extraction. The infant weighed 3700 g and had Apgar scores of 3, 5, and 7 at 1, 5, and 10 minutes, respectively. The infant developed respiratory distress at birth and was assessed by the neonatal resuscitation team. After initial improvement, the infant was admitted to the neonatal intensive care unit for 3 days for monitoring and assessment of oxygen requirements. The available documentation did not identify suspected or confirmed infection, and no congenital anomalies were identified before discharge.

## DISCUSSION

This case series describes 3 pregnancies with brief postconception exposure to JAKi in patients with rheumatic disease (Table). All exposures occurred in the first trimester and were stopped once pregnancy was recognised. All pregnancies resulted in live births, and no congenital anomalies were identified.

**Table:** Clinical summary of pregnancies with postconception JAK inhibitor exposure

Case	Diagnosis	JAK inhibitor exposure	Timing of exposure	Action after pregnancy recognition	Maternal course	Gestational age/delivery	Birth weight	Neonatal outcome
1	Psoriatic arthritis; polycystic kidney disease	Upadacitinib 15 mg daily	16 d postconception	Stopped; no alternative immunosuppression during pregnancy	No clinically documented flare during pregnancy; postpartum flare at 3 mo	37 + 6 wks; elective caesarean	3160 g	Apgar scores 9 and 9 at 1 and 5 min; no congenital anomalies identified
2	Seronegative inflammatory arthritis; ulcerative colitis; CNS demyelinating lesions	Upadacitinib 15 mg daily	13 d postconception	Stopped; later prednisone, ixekizumab trial, then certolizumab	Second-trimester inflammatory arthritis flare; improved with certolizumab	39 wks; elective caesarean	3033 g	Apgar scores 9 and 9 at 1 and 5 min; no congenital anomalies identified
3	Rheumatoid arthritis	Tofacitinib 5 mg twice daily	30 d postconception	Stopped; hydroxychloroquine started	Clinically controlled arthritis during pregnancy	39 wks; emergency caesarean for arrest of descent, complicated by difficult fetal extraction	3700 g	Apgar scores 3, 5, and 7 at 1, 5, and 10 min; respiratory distress at birth; NICU admission for 3 d for monitoring and assessment of oxygen requirements; no infection documented or congenital anomalies identified

CNS, central nervous system; JAK, Janus kinase; NICU, neonatal intensive care unit.

The findings align with a small but growing literature describing inadvertent JAKi exposure in pregnancy, including reports involving tofacitinib, baricitinib, and upadacitinib [[7], [8], [9], [10], [11]]. Published clinical-trial and postmarketing data remain difficult to interpret because exposures are heterogeneous, outcomes are incompletely captured, and comparator groups are generally absent [[7], [8], [9], [10], [11]]. Confounding by underlying disease activity, comorbidities, and concomitant medication further limits causal inference.

The clinical value of these cases is not that they establish JAKi safety in pregnancy. Rather, they provide real-world detail relevant to counselling after inadvertent early exposure. In such situations, counselling should be nondirective, should acknowledge limited data, and should separate the risks of brief early exposure from the unknown risks of continued use later in pregnancy [5,6].

This report has important limitations. The sample size is small, only early first-trimester exposures were observed, and infant follow-up was limited to the early postnatal period. The registry did not include systematic longitudinal paediatric follow-up after maternal rheumatology care returned to the patients’ primary rheumatologists. Consequently, vaccine responses, subsequent infection frequency, and developmental milestones could not be assessed. The absence of these data should not be interpreted as evidence that no such events occurred. Disease activity was assessed clinically in routine care, and standardised disease activity indices were not consistently available. Longer-term developmental and immune outcomes cannot be inferred. Prospective registry studies with standardised exposure windows, comparator groups, and longitudinal infant follow-up are needed.

In summary, 3 pregnancies with inadvertent early exposure to upadacitinib or tofacitinib resulted in live births without identified congenital anomalies. These observations are reassuring for counselling after brief first-trimester exposure, but they should not be interpreted as evidence that JAKi are safe to continue during pregnancy.

## CRediT authorship contribution statement

**Ainsley Needham:** Writing – review & editing, Writing – original draft, Visualization, Data curation. **Vienna Cheng:** Writing – review & editing, Writing – original draft. **Jeremiah Tan:** Writing – review & editing, Writing – original draft, Project administration, Conceptualization. **Mary A. De Vera:** Supervision, Investigation. **Neda Amiri:** Writing – review & editing, Writing – original draft, Validation, Supervision, Methodology, Investigation, Data curation, Conceptualization.

## Competing interests

The authors declare they have no competing interests.
